# The health conditions and the health care consumption of the uninsured

**DOI:** 10.1186/s13561-016-0137-z

**Published:** 2016-12-07

**Authors:** Marco A. Castaneda, Meryem Saygili

**Affiliations:** Department of Social Sciences, The University of Texas at Tyler, 3900 University Blvd, Tyler, TX 75799 USA

**Keywords:** Health insurance, Health conditions, Health care consumption, I10, I13

## Abstract

This paper investigates the difference in the health conditions and the health care consumption of uninsured individuals as compared to individuals with private insurance, using a nationally representative data set of inpatient hospital admissions from the US. In line with the previous literature, our results indicate that uninsured individuals are, on average, in worse health conditions. However, if we compare individuals within the same diagnosis category, the uninsured are actually healthier, with a lower number of chronic conditions and a lower risk of mortality. This indicates that the uninsured are admitted to the hospital only for more serious conditions. In addition, our results show that uninsured individuals consume less health care. In particular, conditional on being admitted to a hospital and controlling for health conditions, the uninsured have lower total charges, fewer procedures, and a higher mortality rate.

## Background

The Affordable Care Act is the most comprehensive reform of the U.S. healthcare system in the last decades and, when fully implemented, it is expected to have a substantial impact on the healthcare system. Moreover, a key motivation for the reform was to provide coverage to the millions of Americans without health insurance. However, there is no agreement in the literature about the health conditions of the uninsured or about how the reform will affect health care costs. Therefore, the purpose of this paper is to provide a more comprehensive analysis of the health conditions and the health care consumption of uninsured individuals as compared to individuals with private insurance.

As in any other insurance market, there are important information problems in the health insurance market. First, the adverse selection problem predicts that individuals with a higher probability of requiring medical care are more likely to buy health insurance. This prediction would imply that the uninsured are healthier compared to the insured. However, the health condition of an individual is not the only determinant of whether or not an individual buys health insurance. The other key factor is income, as individuals with low incomes may not be able to afford health insurance. Therefore, it is not clear if the individuals with no insurance are in better or worse health conditions than the individuals with health insurance.

In addition, there may be a two-way interaction between health conditions and insurance status. The existing literature indicates many individuals without health insurance are not getting the health care required to treat existing conditions and to keep existing conditions from becoming more serious. For instance, uninsured individuals are less likely to get preventive care such as diagnostic tests and are more likely to be admitted to a hospital for conditions that do not require hospitalization if treated in a timely way [14]. The existing evidence concerning the health conditions of the uninsured is limited, as it is derived from the analysis of survey data [6]^1^ or from the analysis of particular conditions such stroke [13].

Second, the moral hazard problem implies that individuals with health insurance consume more health care. There are many studies that show health insurance increases health care consumption. For instance, in the classic RAND Health Insurance Experiment, individuals with more generous health insurance plans had higher health care expenditures [2]. In a similar study, based on the Oregon Health Insurance Experiment, the authors conclude that individuals with health insurance had higher health care utilization [8].

Some researchers, on the other hand, argue that health insurance may alter the type of care individuals get by increasing primary and preventive care and decreasing the use of the Emergency Department (ED), which is quite costly. For instance, Kolstad and Kowalski [9] and Miller [11] investigate the effects of Massachusetts’s health care reform and show that the reform, which decreased the number of uninsured individuals, resulted in a decrease in the number of Emergency Department visits and a decrease in the number of hospital admissions originating in the Emergency Department.

In this paper we use the National Inpatient Sample (NIS) database, which is hospital inpatient administrative data. There are some advantages and disadvantages associated with using this database. A disadvantage of using inpatient administrative data is that we only observe individuals who are admitted to the hospital. Therefore, we investigate the health conditions and health care consumption of the uninsured, conditional on being admitted to the hospital. Given the nature of the data, we will not be able to say much about adverse selection, although we present some indirect evidence. In particular, conditional on being admitted to a hospital, there are systematic differences in the distribution over health conditions between the insured and the uninsured.

A key advantage of using inpatient administrative data is that an inpatient record provides comprehensive information about the health conditions and the health care consumption of an individual. Therefore, the question we will consider is whether individuals with no insurance consumes less health care than individuals with private insurance, conditional on being admitted to a hospital and after carefully controlling for the health conditions of the individuals. As has been noted in the literature, it can be challenging to identify the causal effect of having health insurance on health care consumption because insured and uninsured individuals may have different characteristics. Hence, our identification strategy is based on carefully controlling for health conditions, essentially comparing the health care consumption of individuals with the same set of diagnoses.^2^

Our analysis reveals that uninsured individuals are, *on average*, in worse health conditions. In particular, uninsured patients have a higher number of diagnoses and chronic conditions and a higher severity of illness and risk of mortality. However, once we control for the primary diagnosis, the uninsured are actually in better health conditions. This indicates a clear selection bias, where the uninsured are more likely to be admitted to the hospital for more serious health conditions. Moreover, uninsured individuals are considerably more likely to be admitted to the hospital through the Emergency Department, and they have higher mortality rates. Surprisingly, the higher mortality rates for the uninsured do not go away when we control for health conditions, including the full set of diagnoses. We conjecture that the quality and/or intensity of the health care they receive is potentially different from the insured. In fact, our results show that, conditional on being admitted to the hospital and after carefully controlling for health conditions, uninsured individuals have lower total charges, receive fewer procedures, and are less likely to get a major procedure or an operating room procedure.

## Related literature

The related existing literature can be organized in terms of the health conditions and the health care consumption of the uninsured, as compared to individuals with insurance. As mentioned previously, there are important information problems in the market for health insurance, which make it challenging to identify the causal effect of health insurance on the consumption of health care. For instance, if the less healthy individuals are more likely to purchase health insurance, it may be difficult to identify the health insurance effect without carefully controlling for the health conditions of an individual. For this reason, several approaches have been used in the literature, including randomized experiments and natural experiments resulting from policy decisions.^3^

The best well-known randomized experiments are the RAND Health Insurance Experiment and the Oregon Health Insurance Experiment. The two experiments investigate the effect of health insurance on the consumption of health care, but there are some important differences. In the RAND experiment, every individual in the study was randomly given a health insurance plan, where the plans had various combinations of deductible and coinsurance rate (but the plans had a relatively low maximum for out-of-pocket expenditures). The study investigated how the generosity of the plan, in terms of its cost sharing structure, affected health care expenditures [2]. A particular focus of the RAND experiment was to estimate the out-of-pocket price elasticity of demand for health care expenditures, but the more general results showed that individuals respond to out-of-pocket prices when making health care decisions.

The Oregon experiment involved the use of a lottery to expand Medicaid access to many uninsured low-income individuals. In contrast to the RAND experiment, in which individuals were randomly assigned to multiple insurance plans with marginal variations in cost sharing, in the Oregon experiment individuals were essentially randomly assigned to one health insurance plan (Medicaid) or no health insurance plan. Therefore, the Oregon experiment should provide a better estimate of the effect of health insurance on the consumption of health care, for the particular insurance plan and population in the study. The key results of the study indicate that individuals who were selected by the lottery had substantially higher health care utilization and better self-reported health [8]. In terms of health care utilization, the analysis of the Oregon experiment uses hospital administrative data and some of the same variables used in this paper, including total charges and the number of procedures performed.

A number of studies have used natural experiments resulting from policy decisions. For instance, Antwi et al. [1] use a difference-in-difference approach analysis to investigate the effect of health insurance coverage on hospital admissions based on the provision in the Affordable Care Act that extends the time young individuals are allowed to remain in the health insurance plan of their parents. They estimate that having insurance increases the number of hospital admissions but has no effect on the “intensity of treatment”, where the intensity of treatment includes total charges and the number of procedures performed. Their analysis related to the intensity of treatment is similar to our analysis in this paper related to health care consumption, but our results show that the uninsured have lower total charges and fewer procedures performed. The difference in the results may be explained by noting that the analysis in Antwi et al. [1] includes only young adults.

In addition, Miller [11] investigates the effects of the health care reform in Massachusetts and concludes that the reform, which decreased the number of uninsured individuals, decreased the number of visits to the emergency department, with most of the decrease coming from non-emergency visits. In a similar study, Kolstad and Kowalski [9] investigate the effects of the health care reform in Massachusetts using hospital admissions. Their results indicate that the reform resulted in a decrease in the number of uninsured and a decrease the number of hospital admissions originating from the emergency department. This is consistent with our results, which show that uninsured individuals are more likely to be admitted to the hospital through the emergency department.

More closely related to our work, Doyle [7] investigates the effects of being uninsured on health care consumption and health outcomes by looking at hospital admissions induced by automobile accidents. The interpretation is that automobile accidents act as a random health shock, which allows the researcher to identify the causal effect of being uninsured. The key results of the paper indicate that the uninsured consume 20% less health care and have a substantially higher mortality rate. The results in Doyle [7] are similar to the results presented in this paper but our identification strategy is different, we look at more measures of health care consumption, and we use a much larger and nationally representative sample. In particular, our identification strategy is based on carefully controlling for health conditions, essentially comparing the health care consumption of individuals with the same set of diagnoses. In addition, our measures of health care consumption include total charges and length of stay as well as the total number of procedures, the number of major procedures, and an indicator for an operating room procedure.

Finally, a number of studies have used survey data to investigate the health conditions of the uninsured. For instance, Kronick [10] uses data for multiple years from the National Health Interview Survey to investigate the relationship between being uninsured and the risk of subsequent mortality, as compared to individuals with employer-provided health insurance. The key result indicates that, after controlling for health conditions and health behaviors, being uninsured has no effect on the risk of subsequent mortality. In addition, Polsky et al. [12] uses data from the Health and Retirement Study in a quasi-experimental study to investigate whether enrollment in Medicare has an effect on the health of older and previously uninsured individuals. The study concludes that enrolling in Medicare has only a small and not statistically significant effect on the health of a previously uninsured individual. These results differ from the results presented in this paper, as we show that uninsured individuals have higher mortality rates after controlling for health conditions.

## Data and descriptive statistics

The data used in this study are obtained from the Healthcare Cost and Utilization Project (HCUP) National Inpatient Sample (NIS) database for the year 2011. The data approximates a 20% sample of U.S. community hospitals^4^ and includes all inpatient stays for over 1000 hospitals from 46 states. This database is particularly helpful for our study because it provides a comprehensive description of the medical conditions of the patients and the medical procedures performed, in addition to information on the type of insurance and total charges.

Table 1 describes the initial distribution of admissions by primary expected payer, which we refer to as the type of insurance. We restrict attention to a comparison of individuals in the categories “Self-Pay” and “Private Insurance”. The observations where the primary expected payer is “Self-Pay” are referred to as “Uninsured” and the observations where the primary expected payer is “Private Insurance” are referred to as “Insured”. The reason for restricting attention to observations where the primary expected payer is self-pay or private insurance is that uninsured individuals are not eligible for Medicare or Medicaid, and therefore we think of uninsured individuals as belonging to the same group as individuals with private insurance. In other words, if uninsured individuals are required to have insurance, they would have to purchase private insurance. Therefore, they are likely to behave more like individuals with private insurance rather than individuals with Medicare or Medicaid.^5^

**Table 1:** Distribution of admissions by type of insurance

	Frequency	Percent	Cumulative
Medicare	3,184,258	39.69	39.69
Medicaid	1,572,766	19.60	59.29
Private	2,549,377	31.77	91.06
Self-Pay	391,615	4.88	95.94
No Charge	38,367	0.48	96.42
Other	264,685	3.30	99.72
.	22,522	0.28	100.00
Total	8,023,590	100.00	

### Control variables

The key independent variables in the analysis include insurance status and variables describing the health condition of an individual (DIAGNOSIS variables). Each inpatient record includes a “primary” diagnosis and up to 24 “secondary” diagnoses. We use the Clinical Classification System (CCS) to describe the different diagnoses. In this classification system, there are a total of 264 different diagnoses. In our analysis, we will control for the primary diagnosis (PRIMARY DIAGNOSIS variable) and for the full diagnosis vector (DIAGNOSIS VECTOR variable), which includes the primary diagnosis and the secondary diagnoses. Table 2 illustrates the nature of the diagnosis information. In this situation, all three patients have the same primary diagnosis, but they have different health conditions as indicated by the secondary diagnoses. In our analysis of the health conditions of the uninsured, we control only for the primary diagnosis. In our analysis of the health care consumption of the uninsured, we control for the full diagnosis vector.

**Table 2:** Examples of primary and secondary diagnoses in a patient’s record

	Patient 1	Patient 2	Patient 3
Primary diagnosis	100. Acute Myocardial Infarction	100. Acute Myocardial Infarction	100. Acute Myocardial Infarction
Secondary diagnoses	101. Coronary Atherosclerosis	101. Coronary Atherosclerosis	131. Respiratory Failure
	98. Essential Hypertension	203. Osteoarthritis	
	53. Disorders of Lipid Metabolism		
	49. Diabetes		

In addition, we control for individual characteristics (such as INCOME, GENDER, RACE, and AGE) and hospital characteristics (such as OWNERSHIP status, TEACHING status, and LOCATION). Moreover, in our preferred specifications we include hospital dummy variables in place of hospital characteristics.

Table 3 describes the distribution of admissions by insurance status and individual demographic characteristics. The variable GENDER is a binary variable equal to one for “female” admissions. For the uninsured, the fraction of male admissions is higher than the fraction of female admissions. The opposite is true for insured admissions, where the fraction of male admissions is considerably lower than the fraction of female admissions. The variable RACE consists of “White”, “Black”, “Hispanic”, and “Other”. There are some important differences across race. Blacks and Hispanics have much higher fractions of uninsured admissions, while Whites have the smallest fraction of uninsured admissions. The variable INCOME gives the national quartile of the median household income in the patient ZIP code. As expected, individuals in areas with the lower income quartiles are more likely to be uninsured, while individuals in areas with the higher income quartile are more likely to have private insurance.

**Table 3:** Distribution of admissions by insurance status and individual characteristics

	Insured	Uninsured	Total
All	86.68	13.32	100.00
Gender			
Male	82.82	17.18	100.00
	40.22	54.29	42.09
Female	89.49	10.51	100.00
	59.78	45.71	57.91
Race			
White	89.14	10.86	100.00
	70.87	54.39	68.61
Black	77.25	22.75	100.00
	11.29	20.93	12.61
Hispanic	79.00	21.00	100.00
	10.45	17.49	11.41
Other	86.63	13.37	100.00
	7.40	7.19	7.37
Income			
Quartile 1	77.60	22.40	100.00
	19.45	37.23	21.78
Quartile 2	84.88	15.12	100.00
	22.09	26.09	22.61
Quartile 3	89.29	10.71	100.00
	28.79	22.90	28.01
Quartile 4	93.46	6.54	100.00
	29.68	13.78	27.59

The numbers in the table indicate the *row* percentage and the *column* percentage

Table 4 shows the distribution of admissions by insurance status and hospital characteristics. The “OWNERSHIP” variable is a binary variable equal to one if the hospital is a for-profit hospital. Not-for-profit hospitals include not-for-profit private hospitals as well as public hospitals. The “LOCATION” variable is a dummy variable equal to one if the hospital is located in an urban area. Finally, the “TEACHING” variable is another binary hospital characteristic, which equals one if the hospital is a teaching hospital. Teaching hospitals have a residency program approved by the American Hospital Association or have a membership in the Council of Teaching Hospitals. A large majority of insured and uninsured patients are admitted to not-for-profit hospitals, but the fraction of uninsured patients is slightly higher in for-profit hospitals than in not-for-profit hospital. The shares of admissions at teaching and non-teaching hospitals are approximately equal, but uninsured patients are slightly more likely to go to non-teaching hospitals. The majority of patients are admitted to urban hospitals, with the uninsured being slightly more likely to show up in rural hospitals.

**Table 4:** Distribution of admissions by insurance status and hospital characteristics

	Insured	Uninsured	Total
	86.68	13.32	100.00
Ownership			
For-profit	83.74	16.26	100.00
	12.42	16.01	12.89
Not-for-profit	87.38	12.62	100.00
	87.58	83.99	87.11
Teaching			
Teaching	87.78	12.22	100.00
	50.75	46.93	50.25
Not-teaching	86.04	13.96	100.00
	49.25	53.07	49.75
Location			
Urban	87.34	12.66	100.00
	91.88	88.45	91.43
Rural	82.37	17.63	100.00
	8.12	11.55	8.57

The numbers in the table indicate the *row* percentage and the *column* percentage

### Health conditions variables

Table 5 presents summary statistics for the health condition variables. The variable *Number of Diagnoses* gives the number of diagnoses in the patient’s record. Each diagnosis is categorized as “chronic” or “non-chronic”. A chronic condition is a condition lasting for a year or longer and which meets at least one of the following conditions: (a) it places limitations on self-care, independent living, and social interactions and (b) it results in the need for ongoing medical care. Chronic conditions include conditions such as diabetes, hypertension, and many forms of mental illness. Non-chronic conditions include conditions such as infections, pregnancy, and physical injury. The variable *Number of Chronic Conditions* gives the number of diagnoses categorized as chronic. As illustrated in the table, compared to individuals with private insurance, uninsured individuals have a higher average number of diagnoses and a higher average number of chronic conditions.

**Table 5:** Summary statistics for health conditions variables by insurance status

	Insured	Uninsured	Difference	Percentage difference	All
	86.68	13.32			100.00
Number of diagnoses	6.661 (4.906)	7.068 (4.778)	0.407*** (0.115)	06.11%	6.715 (4.891)
Number of chronic conditions	2.740 (2.931)	3.078 (2.642)	0.338*** (0.0622)	12.33%	2.785 (2.897)
Severity of illness	1.775 (0.857)	1.901 (0.868)	0.126*** (0.0166)	07.10%	1.792 (0.860)
Risk of mortality	1.353 (0.727)	1.417 (0.777)	0.0640*** (0.0118)	04.73%	1.361 (0.734)
Died in hospital	0.0099 (0.099)	0.0140 (0.117)	0.00416*** (0.000858)	41.41%	0.0104 (0.101)
Emergency department	0.353 (0.478)	0.675 (0.469)	0.321*** (0.0109)	91.22%	0.396 (0.489)

The numbers in the table indicate the *mean* and the *standard deviation* in parenthesis. The test refers to the difference in means between the insured and the uninsured

***p < 0.01, **p < 0.05, *p < 0.1

The variable *Severity of Illness* expresses the extent of physiologic decomposition or organ system loss of function and has four subclasses: (1) Minor loss of function, (2) Moderate loss of function, (3) Major loss of function, (4) Extreme loss of function. The presence of multiple conditions in combination with the primary diagnosis determines the severity of illness. An increase in severity of illness reflects increased difficulty and costs involved in treating the patient. On average, uninsured patients have a higher score for severity of illness. Similarly, the variable *Risk of Mortality* indicates the assessed likelihood of dying based on the diagnosis information and includes the following classes: (1) Minor likelihood of dying, (2) Moderate likelihood of dying, (3) Major likelihood of dying, and (4) Extreme likelihood of dying. On the other hand, the binary variable *Died in Hospital* indicates whether a patient actually died in the hospital. Uninsured individuals have a higher assessed risk of mortality and a higher mortality rate.

Finally, the variable *Emergency Department* is a binary variable equal to one if the admission originated in the Emergency Department of the hospital. This variable is based on the “Emergency Department Service” indicator, which is equal to one when the admission record indicates (1) the Emergency Department as the admission source, or (2) an Emergency Department revenue code, or (3) a positive Emergency Department charge, or (4) an Emergency Department procedure code. As illustrated in the table, compared to individuals with insurance, uninsured individuals are considerably more likely to go through the Emergency Department of the hospital.

### Health care consumption variables

The key dependent variables used in the analysis of health care consumption include total charges and length of stay, as well as other outcome measures such as the number of medical procedures performed, the number of major procedures performed, and an indicator variable for a major operating room procedure.

The variable *Total Charges* shows the hospital total charges in dollars for the admission and the variable *Length of Stay* gives the length of stay in days. The total charges consist of “hospital” charges and “professional” charges. The hospital charges include charges for the room and nursing care and charges for services like laboratory tests, medications, and operating room charges. The professional charges are the charges of the attending and consulting physicians. For our purpose of measuring health care consumption, total charges are a reasonable measure because the total charges are based on the procedures performed (which vary across individuals) and the hospital list prices (which do not vary across individuals or type of insurance).^6^ This is consistent with the use of total charges in previous research, where total charges are interpreted “as a price-weighted summary of treatment” [8].

A characterization of the number and types of procedures performed is contained in the variable PRCLASS, which contains the number of procedures in each of the following classes: (1) minor diagnostic (non-operating room procedures such as CT Scan of Head or Diagnostic Cardiac Catheterization), (2) minor therapeutic (non-operating room procedure such as Respiratory Intubation or Circumcision), (3) major diagnostic (operating room procedure such as Laparoscopy or Biopsy of Liver), and (4) major therapeutic (operating room procedure such as Coronary Artery Bypass or Cesarean Section). The variable *Number of Procedures* sums all types of procedures performed. The variable *Number of Major Procedures* is constructed to include major diagnostic and major therapeutic procedures only. Finally, the variable *Operating Room Procedure* is a binary variable equal to one if a major operating room procedure is performed.

Table 6 presents summary statistics for the health care consumption variables. The average total charges for uninsured individuals are lower than the average total charges for insured individuals. The average number of procedures is considerably higher for individuals with insurance. In addition, insured individuals have a higher number of major procedures and they are more likely to get an operating room procedure. The average length of stay is only slightly longer for uninsured patients.

**Table 6:** Summary statistics for health care consumption variables by insurance status

	Insured	Uninsured	Difference	Percentage difference	Total
	86.68	13.32			100.00
Total charges	32,320 (62,650)	29,419 (50,223)	−2901*** (990.9)	−08.98%	31,923 (61,107)
Length of stay	3.850 (5.966)	3.909 (5.846)	0.0587 (0.0909)	01.53%	3.858 (5.950)
Number of procedures	1.738 (2.011)	1.332 (1.970)	−0.406*** (0.0356)	−23.36%	1.683 (2.010)
Number of major procedures	0.606 (1.035)	0.354 (0.858)	−0.251*** (0.0116)	−41.58%	0.572 (1.018)
Operating room procedure	0.377 (0.485)	0.221 (0.415)	−0.155*** (0.00569)	−41.38%	0.355 (0.479)

The numbers in the table indicate the mean and the standard deviation. The test refers to the difference in means between the insured and the uninsured

***p < 0.01, **p < 0.05, *p < 0.1

## Methods and Results

In this section, we present the main results of the paper. First, we provide some results related to the health conditions of the uninsured. Then, we report the results related to the health care consumption of the uninsured as compared to individuals with private insurance.

### Health conditions variables

The measures we use for health conditions include the number of diagnoses, number of chronic conditions, severity of illness, and risk of mortality. For each health condition variable, we estimated an equation of the following form

$$ {Y}_{ij}={\beta}_0+{\beta}_1\kern0.1em  Uninsure{d}_i+{\beta}_2{X}_i+{\delta}_j+{\epsilon}_{ij} $$

where ${Y}_{ij}$ denotes the health condition of individual $i$ in hospital $j$, ${X}_i$ is a vector of individual demographic characteristics, and ${\delta}_j$ represents hospital fixed effects. The individual characteristics contain demographic variables as well as the primary diagnosis. For robustness, we additionally estimate the previous equation using hospital characteristics and state fixed effects. The standard errors are clustered at the hospital level in all specifications. Hence the estimates of standard errors are accurate even if model standard errors for discharges from the same hospital are correlated. In fact, this is likely to be true as the standard errors that control for within-hospital correlation are several times larger than default standard errors [4].

Table 7 presents the results for the *Number of Diagnoses*. The results are reported for various sets of controls to check the robustness of the results and to illustrate how the results change depending on the set of controls. The estimate in column (1) is simply the difference in the average number of diagnoses between the uninsured and the insured. The uninsured have approximately 0.4 more diagnoses on average (about 6% higher, compared to the insured). The higher number of diagnoses may be an indication of worse health conditions. The estimates in column (2) control for *individual characteristics* and the estimates in column (3) additionally control for *hospital characteristics* and include *state fixed effects*. The estimates are considerably lower and no longer statistically significant, with individual characteristics explaining most of the difference. In particular, individuals with lower incomes have a higher number of diagnoses.^7^ The results for race are mixed, with Blacks having a higher number and Hispanics having a lower number of diagnoses, as compared to Whites. The estimates in column (4) additionally control for the *primary diagnosis*. Here, the estimated effect for the uninsured is negative and statistically significant but small (about 3% lower for the uninsured). In other words, after controlling for the primary diagnosis, the uninsured do not appear to be in worse health conditions. Finally, the results in column (5) control for individual characteristics, primary diagnosis, and *hospital fixed effects*. Including hospital fixed effects allows us to control for unobservable hospital characteristics. The estimates are similar to the previous estimates. Overall, the results indicate that on average uninsured individuals have a higher number of diagnoses, but this result is reversed when controlling for the primary diagnosis. The results are very stable across the different specifications, which highlights the robustness of the results.

**Table 7:** Results for number of diagnoses

	(1)	(2)	(3)	(4)	(5)
Variables	Number of diagnoses	Number of diagnoses	Number of diagnoses	Number of diagnoses	Number of diagnoses
Uninsured	0.407***	0.118	0.103	−0.216***	−0.198***
	(0.115)	(0.105)	(0.0825)	(0.0784)	(0.0349)
Income Q1		0.277***	0.435***	0.301***	0.353***
		(0.0991)	(0.0732)	(0.0685)	(0.0211)
Income Q2		0.196**	0.310***	0.215***	0.300***
		(0.0887)	(0.0774)	(0.0740)	(0.0178)
Income Q3		0.167***	0.217***	0.157***	0.178***
		(0.0532)	(0.0447)	(0.0414)	(0.0121)
Black		0.237***	0.182***	−0.00280	0.102***
		(0.0791)	(0.0541)	(0.0527)	(0.0242)
Hispanic		−0.296***	−0.426***	−0.363***	−0.360***
		(0.109)	(0.0832)	(0.0793)	(0.0373)
Other		−0.313***	−0.387***	−0.374***	−0.288***
		(0.0615)	(0.0572)	(0.0501)	(0.0270)
Female		−0.531***	−0.519***	−0.0235*	−0.0186
		(0.0247)	(0.0252)	(0.0143)	(0.0130)
Age		0.106***	0.107***	0.0985***	0.0991***
		(0.00197)	(0.00197)	(0.00190)	(0.00166)
For-profit			−0.237	−0.226	
			(0.148)	(0.145)	
Urban			0.446***	0.487***	
			(0.151)	(0.144)	
Teaching			0.430***	0.396***	
			(0.125)	(0.121)	
State FE	No	No	Yes	Yes	No
Hospital FE	No	No	No	No	Yes
Primary diagnosis FE	No	No	No	Yes	Yes
Observations	2,940,992	2,572,821	2,536,120	2,536,120	2,572,821
R-squared	0.001	0.247	0.266	0.376	0.419

Robust standard errors in parentheses, clustered at the hospital level

***p < 0.01, **p < 0.05, *p < 0.1

Table 8 contains the results for the *Number of Chronic Conditions*. The results are very similar to the previous results. The uninsured have approximately 0.34 more chronic conditions on average (about 12% higher, compared to the insured). The estimated effect becomes smaller but stays positive when we control for individual characteristics and hospital characteristics. However, if we control for the primary diagnosis, the estimated effect for the uninsured is negative and statistically significant (about 7% lower for the uninsured). That is, if we control for the primary diagnosis, the uninsured actually have a lower number of chronic conditions.

**Table 8:** Results for number of chronic conditions

	(1)	(2)	(3)	(4)	(5)
Variables	Number of chronic conditions	Number of chronic conditions	Number of chronic conditions	Number of chronic conditions	Number of chronic conditions
Uninsured	0.338***	0.0829*	0.0751**	−0.221***	−0.188***
	(0.0622)	(0.0423)	(0.0312)	(0.0304)	(0.0191)
Income Q1		0.285***	0.320***	0.214***	0.208***
		(0.0416)	(0.0321)	(0.0264)	(0.0118)
Income Q2		0.214***	0.238***	0.162***	0.167***
		(0.0388)	(0.0353)	(0.0314)	(0.00900)
Income Q3		0.146***	0.153***	0.117***	0.105***
		(0.0240)	(0.0211)	(0.0164)	(0.00694)
Black		0.178***	0.157***	0.0295	0.0746***
		(0.0339)	(0.0241)	(0.0214)	(0.0136)
Hispanic		−0.300***	−0.334***	−0.188***	−0.174***
		(0.0419)	(0.0343)	(0.0291)	(0.0181)
Other		−0.353***	−0.381***	−0.247***	−0.203***
		(0.0352)	(0.0320)	(0.0225)	(0.0153)
Female		−0.696***	−0.688***	−0.0591***	−0.0573***
		(0.0179)	(0.0192)	(0.00654)	(0.00612)
Age		0.0775***	0.0774***	0.0601***	0.0602***
		(0.000880)	(0.000881)	(0.000980)	(0.000924)
For-profit			0.0356	−0.0243	
			(0.0541)	(0.0477)	
Urban			0.222***	0.204***	
			(0.0670)	(0.0570)	
Teaching			0.161***	0.127***	
			(0.0500)	(0.0447)	
State FE	No	No	Yes	Yes	No
Hospital FE	No	No	No	No	Yes
Primary diagnosis FE	No	No	No	Yes	Yes
Observations	2,940,992	2,572,821	2,536,120	2,536,120	2,572,821
R-squared	0.002	0.399	0.408	0.532	0.552

Robust standard errors in parentheses, clustered at the hospital level

***p < 0.01, **p < 0.05, *p < 0.1

Tables 9 and 10 present the results for the *Severity of Illness* and *Risk of Mortality*, respectively. The results parallel the results from the previous health conditions measures. The uninsured have a higher severity of illness and a higher risk of mortality on average, but the results are reversed when we control for the primary diagnosis. For robustness, we performed a similar analysis using the binary variables *High Severity of Illness* (equal to one if severity of illness equals (3) Major loss of function or (4) Extreme loss of function) and *High Risk of Mortality* (equal to one if risk of mortality equals (3) Major likelihood of dying or (4) Extreme likelihood of dying) and we obtained the same pattern of results.

**Table 9:** Results for severity of illness

	(1)	(2)	(3)	(4)	(5)
Variables	Severity of illness	Severity of illness	Severity of illness	Severity of illness	Severity of illness
Uninsured	0.126***	0.0697***	0.0670***	−0.0205**	−0.0135**
	(0.0166)	(0.0148)	(0.0115)	(0.00920)	(0.00647)
Income Q1		0.0486***	0.0629***	0.0351***	0.0434***
		(0.0130)	(0.00821)	(0.00623)	(0.00370)
Income Q2		0.0333***	0.0467***	0.0269***	0.0386***
		(0.0123)	(0.00971)	(0.00829)	(0.00303)
Income Q3		0.0200***	0.0256***	0.0152***	0.0203***
		(0.00760)	(0.00574)	(0.00432)	(0.00232)
Black		0.0957***	0.0813***	0.0537***	0.0566***
		(0.0118)	(0.00687)	(0.00562)	(0.00370)
Hispanic		−0.0120	−0.0260***	−0.0118*	−0.0112***
		(0.0140)	(0.00865)	(0.00707)	(0.00339)
Other		−0.00968	−0.0223***	−0.00578	−0.00247
		(0.0113)	(0.00847)	(0.00610)	(0.00393)
Female		−0.158***	−0.154***	−0.0389***	−0.0375***
		(0.00367)	(0.00370)	(0.00171)	(0.00164)
Age		0.0129***	0.0130***	0.00961***	0.00981***
		(0.000256)	(0.000241)	(0.000273)	(0.000264)
For-profit			−0.0158	−0.00834	
			(0.0142)	(0.0111)	
Urban			0.0698***	0.0799***	
			(0.0164)	(0.0134)	
Teaching			0.101***	0.0769***	
			(0.0143)	(0.0113)	
State FE	No	No	Yes	Yes	No
Hospital FE	No	No	No	No	Yes
Primary diagnosis FE	No	No	No	Yes	Yes
Observations	2,937,203	2,570,871	2,534,240	2,534,240	2,570,871
R-squared	0.002	0.131	0.144	0.311	0.325

Robust standard errors in parentheses, clustered at the hospital level

***p < 0.01, **p < 0.05, *p < 0.1

**Table 10:** Results for risk of mortality

	(1)	(2)	(3)	(4)	(5)
Variables	Risk of mortality	Risk of mortality	Risk of mortality	Risk of mortality	Risk of mortality
Uninsured	0.0640***	0.0113	0.00788	−0.0291***	−0.0248***
	(0.0118)	(0.01000)	(0.00811)	(0.00520)	(0.00415)
Income Q1		0.0418***	0.0436***	0.0247***	0.0296***
		(0.00890)	(0.00553)	(0.00387)	(0.00327)
Income Q2		0.0272***	0.0318***	0.0185***	0.0254***
		(0.00757)	(0.00548)	(0.00424)	(0.00247)
Income Q3		0.0151***	0.0158***	0.00819***	0.0121***
		(0.00469)	(0.00361)	(0.00259)	(0.00196)
Black		0.0611***	0.0534***	0.0335***	0.0353***
		(0.00752)	(0.00559)	(0.00388)	(0.00257)
Hispanic		0.00964	−0.00347	0.00528	0.00495
		(0.0102)	(0.00662)	(0.00498)	(0.00305)
Other		0.0177**	0.00841	0.00563	0.0112***
		(0.00736)	(0.00664)	(0.00471)	(0.00330)
Female		−0.173***	−0.171***	−0.0591***	−0.0583***
		(0.00309)	(0.00315)	(0.00137)	(0.00132)
Age		0.0117***	0.0117***	0.0112***	0.0112***
		(0.000244)	(0.000235)	(0.000280)	(0.000271)
For-profit			−0.00963	−0.00158	
			(0.0107)	(0.00763)	
Urban			0.0546***	0.0609***	
			(0.0125)	(0.00940)	
Teaching			0.0616***	0.0453***	
			(0.0103)	(0.00755)	
State FE	No	No	Yes	Yes	No
Hospital FE	No	No	No	No	Yes
Primary diagnosis FE	No	No	No	Yes	Yes
Observations	2,937,203	2,570,871	2,534,240	2,534,240	2,570,871
R-squared	0.001	0.148	0.155	0.344	0.352

Robust standard errors in parentheses, clustered at the hospital level

***p < 0.01, **p < 0.05, *p < 0.1

The previous results indicate that the uninsured are in worse health conditions on average but are in better health conditions when controlling for the primary diagnosis. The results are not inconsistent with each other and have an intuitive interpretation. In particular, uninsured individuals are more likely to be admitted to the hospital for more serious conditions, which are associated with a higher number of diagnoses and chronic conditions and a higher severity of illness and risk of mortality. For instance, compared to individuals with private insurance, the uninsured are more likely to be admitted to the hospital for Endocrine, Nutritional, and Metabolic diseases (which includes diabetes) and for (ii) Circulatory System diseases (which includes hypertension and myocardial infarction). These results are consistent with the existing literature and the general perceptions about the health conditions of the uninsured. However, once we compare individuals with the same primary diagnosis, the uninsured are actually in better health condition, a result that does not appear in the literature.

In addition, the existing literature indicates the uninsured are more likely to use the Emergency Department to access the healthcare system [11]. We estimated our basic model using the *Emergency Department* variable, which is a binary variable equal to one if the admission originated in the Emergency Department of the hospital. Unlike the study above, our data includes emergency room visits that result in hospitalization. Hence, we only observe real emergencies. In that sense our estimate provides a lower bound on the difference of emergency department utilization between the insured and the uninsured. Table 11 describes the results. The estimate in column (1) is simply the difference in the fraction of admissions originating in the Emergency Department of the hospital between the uninsured and the insured. Hence, conditional on being admitted to the hospital, the uninsured have a substantially higher probability of having been transferred from the Emergency Department to the hospital (about 90% higher, compared to the insured). The estimated effect remains positive and highly statistically significant in all of the specifications. Using the estimate from our preferred specification in Column (5), which controls for individual characteristics, the full diagnosis vector, and hospital fixed effects, the probability of being admitted to the hospital through the Emergency Department is approximately 38% higher for the uninsured compared to the insured.

**Table 11:** Results for emergency

	(1)	(2)	(3)	(4)	(5)
Variables	Emergency	Emergency	Emergency	Emergency	Emergency
Uninsured	0.321***	0.291***	0.287***	0.144***	0.134***
	(0.0109)	(0.0106)	(0.00949)	(0.00732)	(0.00580)
Income Q1		0.00182	0.0190**	−0.00758	0.00318
		(0.0123)	(0.00853)	(0.00691)	(0.00620)
Income Q2		−0.0103	0.00559	−0.0104*	−0.00253
		(0.0105)	(0.00780)	(0.00605)	(0.00469)
Income Q3		−0.00186	0.0134**	0.00366	0.00280
		(0.00816)	(0.00588)	(0.00439)	(0.00300)
Black		0.106***	0.0924***	0.0643***	0.0604***
		(0.00866)	(0.00685)	(0.00533)	(0.00522)
Hispanic		0.0475***	0.0339***	0.0352***	0.0187***
		(0.0107)	(0.00720)	(0.00499)	(0.00306)
Other		−0.0316***	−0.0329***	0.000951	0.000844
		(0.0101)	(0.00779)	(0.00523)	(0.00318)
Female		−0.105***	−0.105***	−0.0205***	−0.0209***
		(0.00577)	(0.00571)	(0.00147)	(0.00138)
Age		0.00645***	0.00636***	0.000505***	0.000444***
		(0.000234)	(0.000223)	(0.000179)	(0.000168)
For-profit			0.0106	0.00788	
			(0.0137)	(0.0103)	
Urban			−0.00674	0.00869	
			(0.0130)	(0.0112)	
Teaching			−0.0627***	−0.0555***	
			(0.0115)	(0.00924)	
State FE	No	No	Yes	Yes	No
Hospital FE	No	No	No	No	Yes
Diagnosis vector FE	No	No	No	Yes	Yes
Observations	2,940,992	2,572,821	2,536,120	2,536,120	2,572,821
R-squared	0.050	0.158	0.201	0.428	0.460

Robust standard errors in parentheses, clustered at the hospital level

***p < 0.01, **p < 0.05, *p < 0.1

Finally, another indicator of the health conditions of the uninsured frequently mentioned in the literature is the actual mortality rate. Therefore, we estimated our basic model using the variable *Died in Hospital*, which is a binary variable equal to one if the individual died in the hospital. Table 12 describes the results. As before, the estimate in column (1) is the difference in the probability of dying in the hospital between the uninsured and the insured. The uninsured have a substantially higher probability of dying (about 42% higher, compared to the insured). The estimated effect remains positive and highly statistically significant in all of the specifications. The estimate in our preferred specification in column (5), which includes individual characteristics, the full diagnosis vector, and hospital fixed effects, indicates the probability of dying in the hospital is about 35% higher for the uninsured as compared to the insured. This result is surprising because we are controlling for the full diagnosis vector. Of course, the probability of dying depends on the health conditions of a patient as well as on the medical procedures performed. In other words, if an individual has a heart attack, the individual is less likely to die if the appropriate medical procedures are performed. Therefore, an explanation for this result may become more apparent after we look at the differences in the consumption of health care between the insured and the uninsured.

**Table 12:** Results for mortality

	(1)	(2)	(3)	(4)	(5)
Variables	Hospital death	Hospital death	Hospital death	Hospital death	Hospital death
Uninsured	0.00416***	0.00247***	0.00237**	0.00320***	0.00350***
	(0.000858)	(0.000946)	(0.000960)	(0.000935)	(0.000983)
Income Q1		0.00195***	0.00188***	0.00127**	0.000428
		(0.000683)	(0.000521)	(0.000496)	(0.000291)
Income Q2		0.000473	0.000814**	0.000326	0.000027
		(0.000552)	(0.000389)	(0.000354)	(0.000240)
Income Q3		−0.000250	−0.0000215	−0.000209	−0.000199
		(0.000424)	(0.000333)	(0.000303)	(0.000208)
Black		0.000616	0.000294	0.000600**	0.000445*
		(0.000443)	(0.000361)	(0.000300)	(0.000239)
Hispanic		0.000548	0.000217	0.000722**	0.000459*
		(0.000430)	(0.000428)	(0.000352)	(0.000264)
Other		0.00250***	0.00188***	0.000430	0.000232
		(0.000462)	(0.000424)	(0.000362)	(0.000305)
Female		−0.00477***	−0.00467***	0.00145***	0.00150***
		(0.000194)	(0.000193)	(0.000176)	(0.000174)
Age		0.000414***	0.000417***	0.000346***	0.000338***
		(0.000016)	(0.000017)	(0.000031)	(0.000031)
For-profit			0.000774	0.00128*	
			(0.000782)	(0.000676)	
Urban			0.000215	−0.00214***	
			(0.000930)	(0.000808)	
Teaching			0.00240***	0.000036	
			(0.000575)	(0.000456)	
State FE	No	No	Yes	Yes	No
Hospital FE	No	No	No	No	Yes
Diagnosis vector FE	No	No	No	Yes	Yes
Observations	2,936,294	2,570,122	2,533,564	2,533,564	2,570,122
R-squared	0.000	0.009	0.010	0.200	0.204

Robust standard errors in parentheses, clustered at the hospital level

***p < 0.01, **p < 0.05, *p < 0.1

### Health care consumption variables

The following results relate to the health care consumption variables, which include total charges, number of procedures, number of major procedures, an indicator of an operating room procedure, and length of stay. We estimate the same basic model as in the previous section, but now the dependent variable refers to the health consumption variables.

Table 13 shows the results for *Total Charges* in dollars. Column (1) indicates that the total charges for the uninsured are about $2900 less on average (about 9% less compared with the insured). The estimates in column (2) control for individual characteristics and the estimates in column (3) additionally control for hospital characteristics and state fixed effects. This increases the gap between the insured and the uninsured, the latter paying about $5000 less on average (about 15% less compared to the insured). The estimates in column (4) additionally controls for the *full diagnosis vector*. This allows us to compare individuals with similar health conditions based on their insurance status. The estimates in column (5) control for individual characteristics, the full diagnosis vector, and hospital fixed effects, which is our preferred set of controls. The estimated effect of being uninsured is consistently negative and statistically significant. The estimate in column (5) indicates the uninsured are charged about $1900 less on average (about 6% less, compared to the insured). Finally, the estimates in column (5) indicate that Blacks and Hispanics have lower total charges (compared to Whites), females have lower total charges (compared to males), and the total charges increase with age. The estimates in column (3) or column (4), which control for hospital characteristics, indicate that total charges are higher in for-profit hospitals, in urban hospitals, and in teaching hospitals.

**Table 13:** Results for total charges

	(1)	(2)	(3)	(4)	(5)	(6)
Variables	Total charges	Total charges	Total charges	Total charges	Total charges	Total charges
Uninsured	−2901***	−5039***	−4431***	−2820***	−1918***	454.5
	(990.9)	(1007)	(633.6)	(455.6)	(275.8)	(302.2)
Income Q2		1345**	1068**	920.1**	614.9***	259.3
		(605.4)	(499.2)	(390.8)	(232.9)	(192.5)
Income Q3		2407**	369.5	1252**	482.4*	184.7
		(1062)	(690.6)	(548.9)	(274.7)	(233.9)
Income Q4		2521	135.9	1340*	92.68	−371.6
		(1652)	(979.6)	(789.5)	(339.3)	(291.1)
Black		1080	241.5	697.1	−1285***	860.6***
		(1097)	(735.8)	(557.1)	(356.1)	(251.8)
Hispanic		5456***	−2167**	−372.9	−1389***	−941.8***
		(1423)	(944.2)	(812.2)	(267.0)	(238.3)
Other		2212*	−994.2	774.2	−302.5	−394.5
		(1263)	(911.5)	(570.3)	(516.1)	(456.0)
Female		−8620***	−8188***	−1030***	−923.0***	−462.6***
		(536.6)	(423.7)	(180.3)	(164.0)	(127.6)
Age		532.4***	522.5***	23.54*	41.17***	124.5***
		(23.26)	(20.19)	(14.14)	(11.79)	(10.05)
For-profit			11,219***	11,342***		
			(2385)	(2060)		
Urban			8833***	5057***		
			(1380)	(1150)		
Teaching			12,919***	7028***		
			(1782)	(1259)		
State FE	No	No	Yes	Yes	No	No
Hospital FE	No	No	No	No	Yes	Yes
Diagnosis vector FE	No	No	No	Yes	Yes	No
Procedure vector FE	No	No	No	No	No	Yes
Observations	2,834,320	2,475,053	2,438,353	2,438,353	2,475,053	2,475,053
R-squared	0.000	0.043	0.079	0.344	0.378	0.509

Robust standard errors in parentheses, clustered at the hospital level

***p < 0.01, **p < 0.05, *p < 0.1

Our previous results on health condition variables show that controlling for primary diagnosis, the uninsured are not in worse health conditions compared to the insured. However, the current estimates include the full set of diagnoses, not just the primary diagnosis. Hence, we conclude that, after controlling for the health conditions of a patient, the uninsured consume less healthcare. This suggests that the uninsured receive fewer procedures and/or less expensive procedures. As a test of this hypothesis, the estimates in Column (6) control for the *full procedure vector* in place of the full diagnosis vector. Once we control for the particular set of procedures that patients get, the difference between the insured and the uninsured disappears (the estimate is considerably smaller and not statistically significant). This supports the idea that the uninsured have lower total charges because they consume less health care.^8^

The results for *Number of Procedures* are reported in Table 14. The results indicate that the uninsured get fewer procedures compared to the insured. The results in column (5), which control for individual characteristics, the full diagnosis vector, and hospital fixed effects, show the uninsured are getting about 8% fewer procedures. The estimated effect of being uninsured on the number of procedures gets smaller in absolute value when we control for the full diagnosis vector. This suggests the uninsured are getting fewer procedures partially because they are in better health conditions. However, even if we control for the full diagnosis vector, the coefficient is still negative and statistically significant. That is, uninsured individuals get a smaller number of procedures when compared to insured individuals with the same diagnosis vector. This supports our claim that total charges are smaller for the uninsured because they consume less health care.

**Table 14:** Results for number of procedures

	(1)	(2)	(3)	(4)	(5)
Variables	Number of procedures	Number of procedures	Number of procedures	Number of procedures	Number of procedures
Uninsured	−0.406***	−0.417***	−0.384***	−0.148***	−0.135***
	(0.0356)	(0.0341)	(0.0234)	(0.0158)	(0.0102)
Income Q2		0.0947***	0.0639***	0.0347**	0.0190**
		(0.0247)	(0.0208)	(0.0173)	(0.00787)
Income Q3		0.0911***	0.00347	−0.00767	0.00890
		(0.0288)	(0.0208)	(0.0142)	(0.00907)
Income Q4		0.135***	0.00725	−0.00313	0.00870
		(0.0432)	(0.0277)	(0.0208)	(0.0106)
Black		−0.105***	−0.185***	−0.0964***	−0.0913***
		(0.0323)	(0.0285)	(0.0210)	(0.0118)
Hispanic		−0.000980	−0.0890***	−0.101***	−0.0531***
		(0.0368)	(0.0294)	(0.0237)	(0.0118)
Other		0.135***	0.0566**	−0.0238	−0.0230*
		(0.0402)	(0.0268)	(0.0182)	(0.0119)
Female		−0.0793***	−0.0637***	−0.153***	−0.153***
		(0.0181)	(0.0164)	(0.00648)	(0.00611)
Age		0.0134***	0.0139***	0.00220***	0.00243***
		(0.000628)	(0.000582)	(0.000437)	(0.000345)
For-profit			−0.0338	−0.00525	
			(0.0594)	(0.0377)	
Urban			0.374***	0.196***	
			(0.0570)	(0.0435)	
Teaching			0.416***	0.240***	
			(0.0563)	(0.0410)	
State FE	No	No	Yes	Yes	No
Hospital FE	No	No	No	No	Yes
Diagnosis vector FE	No	No	No	Yes	Yes
Observations	2,940,992	2,572,821	2,536,120	2,536,120	2,572,821
R-squared	0.005	0.029	0.049	0.372	0.403

Robust standard errors in parentheses, clustered at the hospital level

***p < 0.01, **p < 0.05, *p < 0.1

In addition, these results are consistent with, and help explain, our previous findings regarding total charges. In particular, Black and Hispanic patients are getting a smaller number of procedures compared to Whites, females get fewer procedures compared to males, and the number of procedures performed increases with age. Finally, the results in column (4) indicate that there is no significant difference between for-profit and non-profit hospitals in terms of the number of procedures [5], but as expected patients get more procedures in urban hospitals and in teaching hospitals.

For robustness, we performed a similar analysis using the variables *Number of Major Procedures* and *Operating Room Procedure*. Table 15 presents the results for the number of major procedures and Table 16 show the results for the indicator variable of an operating room procedure. Based on our preferred specification in column (5), the number of major procedures is 18% lower for the uninsured and the probability of getting an operating room procedure is 17% lower for the uninsured compared to the insured. The qualitative implications are the same as for the number of procedures. In particular, uninsured patients get a smaller number of procedures, fewer major procedures, and are less likely to get an operating room procedure.

**Table 15:** Results for number of major procedures

	(1)	(2)	(3)	(4)	(5)
Variables	Number of major procedures	Number of major procedures	Number of major procedures	Number of major procedures	Number of major procedures
Uninsured	−0.251***	−0.255***	−0.251***	−0.119***	−0.110***
	(0.0116)	(0.0113)	(0.00983)	(0.00599)	(0.00481)
Income Q2		0.0379***	0.0384***	0.0195***	0.0121***
		(0.00922)	(0.00875)	(0.00532)	(0.00399)
Income Q3		0.0281**	0.0146*	0.00572	0.00831*
		(0.0114)	(0.00867)	(0.00504)	(0.00477)
Income Q4		0.0409***	0.0331***	0.0157**	0.0108
		(0.0155)	(0.0119)	(0.00736)	(0.00663)
Black		−0.0869***	−0.107***	−0.0706***	−0.0682***
		(0.0112)	(0.0105)	(0.00626)	(0.00628)
Hispanic		−0.0395***	−0.0550***	−0.0593***	−0.0508***
		(0.0131)	(0.00934)	(0.00601)	(0.00447)
Other		−0.0602***	−0.0688***	−0.0421***	−0.0428***
		(0.0105)	(0.00980)	(0.00575)	(0.00537)
Female		−0.0646***	−0.0599***	−0.146***	−0.146***
		(0.00662)	(0.00638)	(0.00589)	(0.00584)
Age		0.00565***	0.00571***	0.00274***	0.00295***
		(0.000263)	(0.000268)	(0.000173)	(0.000148)
For-profit			0.0309	0.0334***	
			(0.0230)	(0.0125)	
Urban			0.123***	0.0807***	
			(0.0202)	(0.0134)	
Teaching			0.144***	0.0835***	
			(0.0185)	(0.0110)	
State FE	No	No	Yes	Yes	No
Hospital FE	No	No	No	No	Yes
Diagnosis vector FE	No	No	No	Yes	Yes
Observations	2,940,992	2,572,821	2,536,120	2,536,120	2,572,821
R-squared	0.007	0.026	0.034	0.362	0.375

Robust standard errors in parentheses, clustered at the hospital level

***p < 0.01, **p < 0.05, *p < 0.1

**Table 16:** Results for operating room procedure

	(1)	(2)	(3)	(4)	(5)
Variables	Operating room procedure	Operating room procedure	Operating room procedure	Operating room procedure	Operating room procedure
Uninsured	−0.155***	−0.158***	−0.156***	−0.0686***	−0.0627***
	(0.00569)	(0.00585)	(0.00581)	(0.00400)	(0.00324)
Income Q2		0.0166***	0.0180***	0.00691***	0.00537***
		(0.00399)	(0.00361)	(0.00214)	(0.00194)
Income Q3		0.0208***	0.0167***	0.00698***	0.00497**
		(0.00512)	(0.00393)	(0.00240)	(0.00229)
Income Q4		0.0278***	0.0264***	0.0119***	0.00681**
		(0.00653)	(0.00543)	(0.00351)	(0.00337)
Black		−0.0467***	−0.0552***	−0.0396***	−0.0352***
		(0.00541)	(0.00474)	(0.00287)	(0.00299)
Hispanic		−0.0189***	−0.0218***	−0.0406***	−0.0353***
		(0.00493)	(0.00410)	(0.00345)	(0.00286)
Other		−0.0264***	−0.0276***	−0.0322***	−0.0314***
		(0.00503)	(0.00492)	(0.00332)	(0.00296)
Female		−0.0739***	−0.0726***	−0.132***	−0.133***
		(0.00413)	(0.00421)	(0.00545)	(0.00544)
Age		0.00142***	0.00144***	0.00170***	0.00181***
		(0.000140)	(0.000142)	(9.19e-05)	(8.62e-05)
For-profit			0.00624	0.0109**	
			(0.0101)	(0.00550)	
Urban			0.0608***	0.0479***	
			(0.00919)	(0.00602)	
Teaching			0.0498***	0.0379***	
			(0.00738)	(0.00471)	
State FE	No	No	Yes	Yes	No
Hospital FE	No	No	No	No	Yes
Diagnosis vector FE	No	No	No	Yes	Yes
Observations	2,940,992	2,572,821	2,536,120	2,536,120	2,572,821
R-squared	0.012	0.025	0.031	0.379	0.389

Robust standard errors in parentheses, clustered at the hospital level

***p < 0.01, **p < 0.05, *p < 0.1

The regression results for *Length of Stay* are displayed in Table 17. The coefficient on insurance status is negative but not statistically significant, indicating that the length of stay for the uninsured is not different from that of the insured. The coefficient on the gender dummy changes sign when we include the full diagnosis vector. Females, on average, have a shorter length of stay, but if we compare different genders with the same diagnosis vector, females tend to have a longer length of stay.^9^ This suggests female patients are in general admitted to hospitals with conditions that require shorter stays. The coefficient on age is positive but becomes insignificant once we control for the diagnosis vector. Controlling for the diagnosis vector, Blacks and Hispanics have a longer length of stay. If the length of stay and the number of procedures (possibly, the type of procedures as well) determine the total charges, then the fact that Black and Hispanic patients cost less despite staying longer implies the intensity of care they receive is substantially less.

**Table 17:** Results for length of stay

	(1)	(2)	(3)	(4)	(5)
Variables	Length of stay	Length of stay	Length of stay	Length of stay	Length of stay
Uninsured	0.0587	−0.134	−0.111	−0.0899	−0.0556
	(0.0909)	(0.0948)	(0.0853)	(0.0652)	(0.0525)
Income Q2		−0.0963**	−0.0767*	−0.0376	0.00722
		(0.0464)	(0.0424)	(0.0370)	(0.0264)
Income Q3		−0.190***	−0.220***	−0.112***	−0.00603
		(0.0650)	(0.0521)	(0.0414)	(0.0255)
Income Q4		−0.200**	−0.248***	−0.120***	−0.0220
		(0.0882)	(0.0564)	(0.0441)	(0.0326)
Black		0.422***	0.303***	0.250***	0.199***
		(0.0631)	(0.0572)	(0.0457)	(0.0425)
Hispanic		−0.0648	−0.171***	0.0545	0.0578**
		(0.0779)	(0.0559)	(0.0388)	(0.0229)
Other		0.171**	0.0795	0.201***	0.177***
		(0.0786)	(0.0578)	(0.0372)	(0.0269)
Female		−0.564***	−0.530***	0.0523***	0.0639***
		(0.0241)	(0.0220)	(0.0145)	(0.0138)
Age		0.0225***	0.0229***	0.00153	0.000481
		(0.00123)	(0.00109)	(0.00127)	(0.000910)
For-profit			0.0908	0.119*	
			(0.107)	(0.0685)	
Urban			0.539***	0.272***	
			(0.0916)	(0.0743)	
Teaching			0.669***	0.202***	
			(0.0851)	(0.0514)	
State FE	No	No	Yes	Yes	No
Hospital FE	No	No	No	No	Yes
Diagnosis vector FE	No	No	No	Yes	Yes
Observations	2,940,954	2,572,789	2,536,088	2,536,088	2,572,789
R-squared	0.000	0.011	0.017	0.339	0.362

Robust standard errors in parentheses, clustered at the hospital level

***p < 0.01, **p < 0.05, *p < 0.1

As a summary, our results from the analysis of the health care consumption variables indicate that uninsured individuals consume less health care. In particular, if we compare insured and uninsured individuals with the same diagnosis vector, who are treated at the same hospital, the uninsured have lower total charges, a smaller number of procedures, and a smaller probability of having an operating room procedure. The difference in the health care consumption of the insured and the uninsured may help explain why the uninsured have a higher mortality rate.

## Discussion and Conclusion

This paper attempts to take a closer look at the health conditions and the health care consumption patterns of the uninsured compared to the insured using a nationally representative inpatient dataset from the US. A key advantage of using inpatient administrative data is that an inpatient record includes comprehensive information about the health conditions and the health care consumption of an individual. As has been noted in the literature, it can be challenging to identify the causal effect of having health insurance because insured and uninsured individuals may have different characteristics. Therefore, our identification strategy is based on carefully controlling for health conditions, essentially comparing the health care consumption of individuals with the same set of diagnoses treated at the same hospital.

First, we find that conditional on being admitted to the hospital the uninsured are, on average, in worse health conditions. This is a result frequently cited in the literature. However, if we control for the primary diagnoses, the uninsured are actually in better health conditions than the insured. Surprisingly, this is not a well-known finding in the literature. These results together imply that uninsured individuals go to the hospital for more serious reasons that are associated with a higher number of chronic conditions and a higher severity of illness and risk of mortality. For instance, compared to individuals with private insurance, the uninsured are more likely to be admitted to the hospital for conditions related to the Circulatory System (such as Acute Myocardial Infarction) but are less likely to be admitted to the hospital for conditions related to the Musculoskeletal System (such as Rheumatoid Arthritis). Therefore, studies that compare health conditions of the uninsured and the insured based on inpatient data can be misleading. They are comparing very sick uninsured individuals, who have no choice but to go the hospital, to the insured, which may include patients with less severe or non-urgent conditions. We do not observe the uninsured with less severe or non-urgent conditions in the data simply because they do not go the hospital.

Second, the uninsured are more likely to be admitted to the hospital through the Emergency Department, and are more likely to die at the hospital. These findings are consistent with the existing literature. These results persist even if we control for the full diagnosis vector. Some researchers argue that the uninsured may choose to seek care in the emergency room because, unlike private physicians, emergency rooms are legally obligated to treat people, even if they cannot pay for services. Our data includes emergency room visits that result in hospitalization. Hence, we only observe real emergencies. In that sense our estimate provides a lower bound on the difference of Emergency Department utilization between the insured and the uninsured. The probability of dying in the hospital is about 35% higher for the uninsured as compared to the insured. If uninsured individuals are more likely to die compared to insured individuals with similar health conditions, this may indicate that the quality or intensity of the health care they receive is potentially different.

Third, again conditional on being admitted to the hospital, the uninsured consume less health care. They have smaller total charges, receive a smaller number of procedures, and are less likely to get an operating room procedure. In our analysis, to control for possible differences in health conditions, we include the full diagnosis vector. In addition, we control for individual characteristics and hospital fixed effects. This implies that when we compare insured and uninsured individuals with the same set of diagnoses, who are treated at the same hospital, the uninsured have lower total charges. The difference disappears when we control for the set of procedures in place of the set of diagnoses. This reveals that the uninsured have lower total charges because they are getting fewer and/or less costly procedures. In this study we only look at the number of procedures. The question of whether the uninsured get alternative and less expensive procedures compared to the insured is left as future research.

The results of this study suggest that providing health insurance coverage to the millions of Americans without health insurance is likely to improve the health conditions of the uninsured but may result in higher healthcare costs. First, the results imply that uninsured individuals go to the hospital for more serious conditions and are considerably more likely to be admitted through the Emergency Department. Therefore, it is reasonable to conjecture that the uninsured individuals are not getting the necessary care in a timely manner and frequently seek care only when their condition deteriorates to the point of requiring hospitalization. In this situation, providing coverage to the uninsured is likely to improve health conditions and may actually lower healthcare costs by providing treatment at an early stage and decreasing the use of emergency care. On the other hand, our results indicate that the uninsured consume less health care. This suggests that providing coverage to the uninsured may increase healthcare cost by increasing the intensity of treatment and by allowing previously uninsured individuals to get care for non-emergency or elective procedures. Finally, the results related to in-hospital mortality rates, combined with our previous results, suggest that providing coverage to the uninsured is likely to lower the in-hospital mortality rates of the uninsured.

## Footnotes

1 The authors use the National Health and Nutritional Examination Survey and conclude that Medicare coverage facilitates outpatient physician care and improves blood pressure control.

2 In general, an individual with health insurance may consume more health care in any given visit to the hospital and may visit the hospital more frequently. We cannot tell if an individual visited the hospital multiple times during the period of analysis. Therefore, our analysis is only a partial test of the “over consumption” effect associated with having health insurance.

3 In addition, Bajari et al. [3] estimate a structural model of health insurance and healthcare consumption using data from a large self-insured employer and report evidence of adverse selection and moral hazard.

4 The universe of U.S. community hospitals is divided into strata using various hospital characteristics and the hospitals in the sample are a stratified probability sample, with sampling probabilities proportional to the number of hospitals in each stratum.

5 This is similar to the sample selection in Kronick [10], who compares the uninsured to individuals with employer-provided health insurance.

6 The total charges are not equal to the “allowed charges,” which are the actual charges to the individual or insurance company. Generally, an insurance company will negotiate prices with a hospital to determine the allowed charges. In addition, the allowed charges for uninsured individuals are frequently considerably lower than the total charges.

7 The income measure is an imprecise measure of the true income of a patient. Moreover, because income is correlated with being uninsured and with health conditions, this may bias the estimated effect of being uninsured towards poor health. However, because the results already show that the uninsured are not in worse health conditions, this actually strengthens the results.

8 For robustness, we performed a similar analysis using the natural log of total charges and obtained very similar results.

9 The coefficient is positive and significant even if we omit maternal admissions.
